# Regulation of Coagulation Factor XI Expression by MicroRNAs in the Human Liver

**DOI:** 10.1371/journal.pone.0111713

**Published:** 2014-11-07

**Authors:** Salam Salloum-Asfar, Raúl Teruel-Montoya, Ana B. Arroyo, Nuria García-Barberá, Amarjit Chaudhry, Erin Schuetz, Ginés Luengo-Gil, Vicente Vicente, Rocío González-Conejero, Constantino Martínez

**Affiliations:** 1 Centro Regional de Hemodonación, University of Murcia, Instituto Murciano de Investigación Biosanitaria Virgen de la Arrixaca, Murcia, Spain; 2 Department of Pharmacology, St. Jude Children's Research Hospital, Memphis, Tennessee, United States of America; Saint Louis University, United States of America

## Abstract

High levels of factor XI (FXI) increase the risk of thromboembolic disease. However, the genetic and environmental factors regulating FXI expression are still largely unknown. The aim of our study was to evaluate the regulation of FXI by microRNAs (miRNAs) in the human liver. *In silico* prediction yielded four miRNA candidates that might regulate FXI expression. HepG2 cells were transfected with miR-181a-5p, miR-23a-3p, miR-16-5p and miR-195-5p. We used mir-494, which was not predicted to bind to *F11*, as a negative control. Only miR-181a-5p caused a significant decrease both in FXI protein and *F11* mRNA levels. In addition, transfection with a miR-181a-5p inhibitor in PLC/PRF/5 hepatic cells increased both the levels of *F11* mRNA and extracellular FXI. Luciferase assays in human colon cancer cells deficient for Dicer (HCT-DK) demonstrated a direct interaction between miR-181a-5p and 3′untranslated region of *F11*. Additionally, *F11* mRNA levels were inversely and significantly correlated with miR-181a-5p levels in 114 healthy livers, but not with miR-494. This study demonstrates that FXI expression is directly regulated by a specific miRNA, miR-181a-5p, in the human liver. Future studies are necessary to further investigate the potential consequences of miRNA dysregulation in pathologies involving FXI.

## Introduction

Although coagulation factor XI (FXI) was discovered nearly 50 years ago [1], its role in pathophysiological conditions is still not fully understood. A wide range of FXI plasma levels has been found in the healthy population [2]. The available functional data on FXI function are confusing, probably reflecting the fact that FXI might be involved not only in haemostasis but also in pathologic processes as inflammation or innate immunity [3], [4]. Epidemiological and animal model studies have associated FXI levels with the risk of thrombotic disease (for review see [5], [6]) or septic survival advantage [7]. On the other hand, FXI deficiency does not usually lead to spontaneous bleeding, but it is associated with an increased risk of bleeding when the haemostatic system is challenged [6], [8]. Moreover, FXI inhibition has been proposed as a novel approach to developing new anti-thrombotic therapies to achieve an improved benefit-risk ratio [9], [10].

In this framework, several groups have been engaged in an intensive study of the influence of genetic and environmental factors on FXI plasma levels in an attempt to understand whether the heterogeneous values found in the healthy population confer a pro- or anti-thrombotic phenotype. Although some of these studies have identified the involvement of common single nucleotide polymorphisms in the structural *F11* gene and alterations in other genes that might indirectly regulate plasma levels of this factor [11]–[13], the molecular mechanisms of FXI regulation are still largely unknown.

MicroRNAs (miRNAs), which are small non-coding RNAs that regulate protein expression [14], have been involved in the regulation of many complex mechanisms or physiological conditions, including the haemostatic system. Available predictive algorithms estimate that a third of the human mRNAs may contain a single or multiple binding sites for miRNAs [15]. As such, a single miRNA can potentially target hundreds of genes, or a single gene could be targeted by many different miRNAs [15]–[18]. However, it has been shown that overexpression of miRNAs only provokes a mild repression of both mRNA [19] and protein [20].

During the last four years, several groups including ours, have evaluated the role of miRNAs in the regulation of haemostasis [21]. Coagulation factors like fibrinogen [22] or tissue factor have been described as interacting with miRNAs, which may have an impact on the thrombotic etiology associated with pathologies such as antiphospholipid syndrome or systemic lupus erythematosus [23]. Recently, PAI-1 [24] and protein S [25] have also been shown to be directly regulated by miRNAs. In the current study, we investigated the potential relevance of miRNAs aiming to discover new elements that may modulate FXI in the liver. *In silico* predictions together with *in vitro* experiments showed that only miR-181a-5p caused a slight although significant decrease both in FXI protein and *F11* mRNA levels. Luciferase assays helped us to demonstrate a direct interaction between miR-181a-5p and 3′ untranslated region (3′UTR) of *F11*. Importantly, *F11* mRNA levels were inversely and significantly correlated with miR-181a-5p levels in 114 healthy livers. This study demonstrates that FXI expression in the human liver is directly regulated by a specific miRNA, miR-181a-5p, opening up new prospects in a better understanding of the pathophysiology of haemostatic diseases where FXI is involved and in the development of miRNA-based therapeutic technologies.

## Results

### A microarray and *in silico* target search yielded four miRNAs that could potentially bind to *F11* mRNA

**Figure 1:**
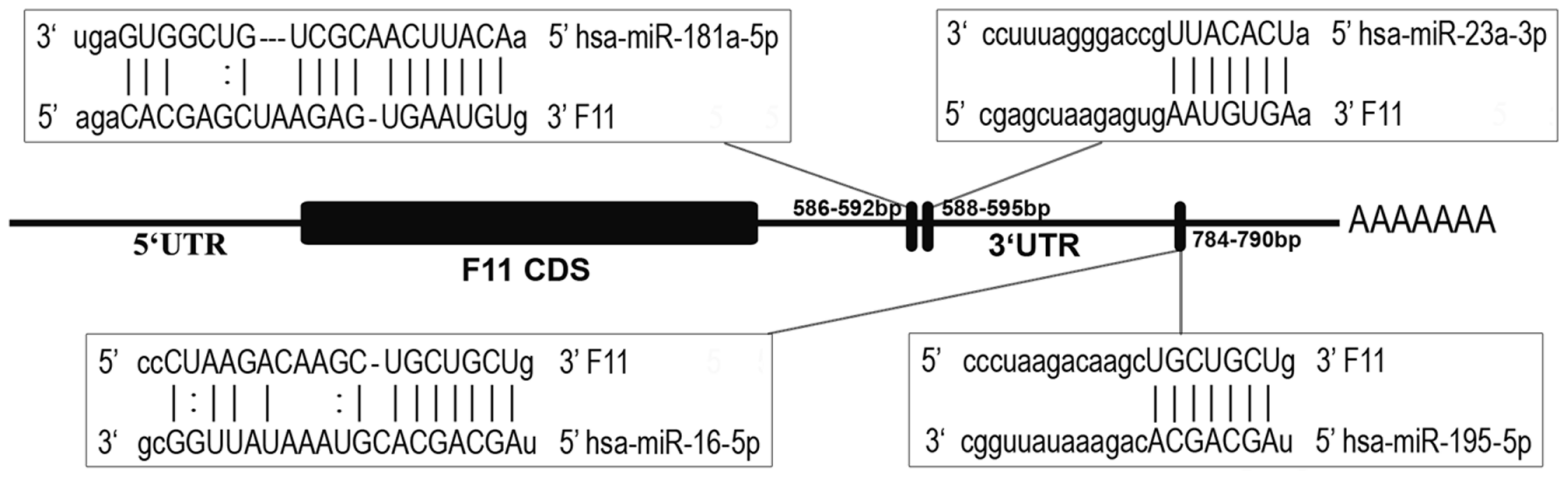
Schematic representation of predicted target sites of miRNAs in *F11* 3′UTR. The predicted binding sites of miR-181a-5p, miR-23a-3p, miR-16-5p, and miR-195-5p are indicated in the *F11* 3′UTR (1,060 bp). The first nucleotide after the stop codon of *F11* is defined as “+1”, and the start- and end-positions of the complementary sequence between *F11* and miRNAs are indicated. Complementarities between the seed region (7 nucleotides) of miRNAs and 3′UTR of *F11* mRNA target site are shown in parentheses. MiR-16-5p and miR-195-5p share the same binding site. MiRNA: mRNA interactions are represented by upper-case letters (provided by mirSVR algorithm).

In order to select miRNAs with the potential to bind to *F11* mRNA, two criteria were established (i) the miRNA expression cut-off in liver had to be >500 arbitrary units (au) (see array in Table S1) and (ii) the miRNA binding had to be anticipated in 4 or more of the prediction algorithms of miRNA targets used (n = 8). Such filtering allowed the selection of four miRNAs: miR-181a-5p (liver expression  = 1233 au; 6 prediction algorithms), miR-23a-3p (liver expression  = 6052 au; 5 prediction algorithms), miR-16-5p (liver expression  = 3513 au; 4 prediction algorithms), and miR-195-5p (liver expression  = 3046 au; 4 prediction algorithms) (see Table 1 and Table S1). Additionally, a negative control miRNA (miR-494; see Table 1), which was not predicted to bind to *F11* mRNA (only 2 prediction algorithms) and with a liver expression >500 au, was also investigated (Table 1). Putative binding sites for miRNAs are shown in Figure 1. Whereas miR-181a-5p and miR-23a-3p bind to two closely located sites, miR-16-5p and miR-195-5p share the same binding site located ∼200 bp downstream of the miR-23a-3p seed (Figure 1).

**Table 1:** *In silico* Prediction Results.

Human miRNA	RNAhybrid^A^	TargetScan^B^	miRSVR^C^	DIANAmT^D^	miRDB^D^	PITA^D^	RNA22^D^	PICTAR5^D^	Total prediction algorithms	Liver Expression (arbitrary units)
**miR-181a-5p**	**1 (−19.70)**	**1 (−0.05)**	**1 (−0.36)**	**1**	**1**	**1**	**-**	**-**	**6**	**1233**
**miR-23a-3p**	**1 (−14.70)**	**1 (−0.07)**	**1 (−0.67)**	**1**	**-**	**1**	**-**	**-**	**5**	**6052**
**miR-16-5p**	**1 (−15.00)**	**1 (−0.13)**	**1 (−0.13)**	**1**	**-**	**-**	**-**	**-**	**4**	**3513**
**miR-195-5p**	**1 (−8.00)**	**1 (−0.13)**	**1 (−0.14)**	**1**	**-**	**-**	**-**	**-**	**4**	**3046**
**miR-494**	**-**	**-**	**1 (−0.03)**	**-**	**-**	**1**	**-**	**-**	**2**	**1395**

The presence of the selected miRNA in the target prediction tool is recognized in the table by number “1”.

A: RNA hybrid minimum free energy (MFE; kcal/mol) [42], B: TargetScan (context + score) [43], C: miRSVR score[44], D: algorithms included in miRWalk.

### *In vitro* studies suggested miR-181a-5p as a direct inhibitor of FXI and *F11* mRNA expression

**Figure 2:**
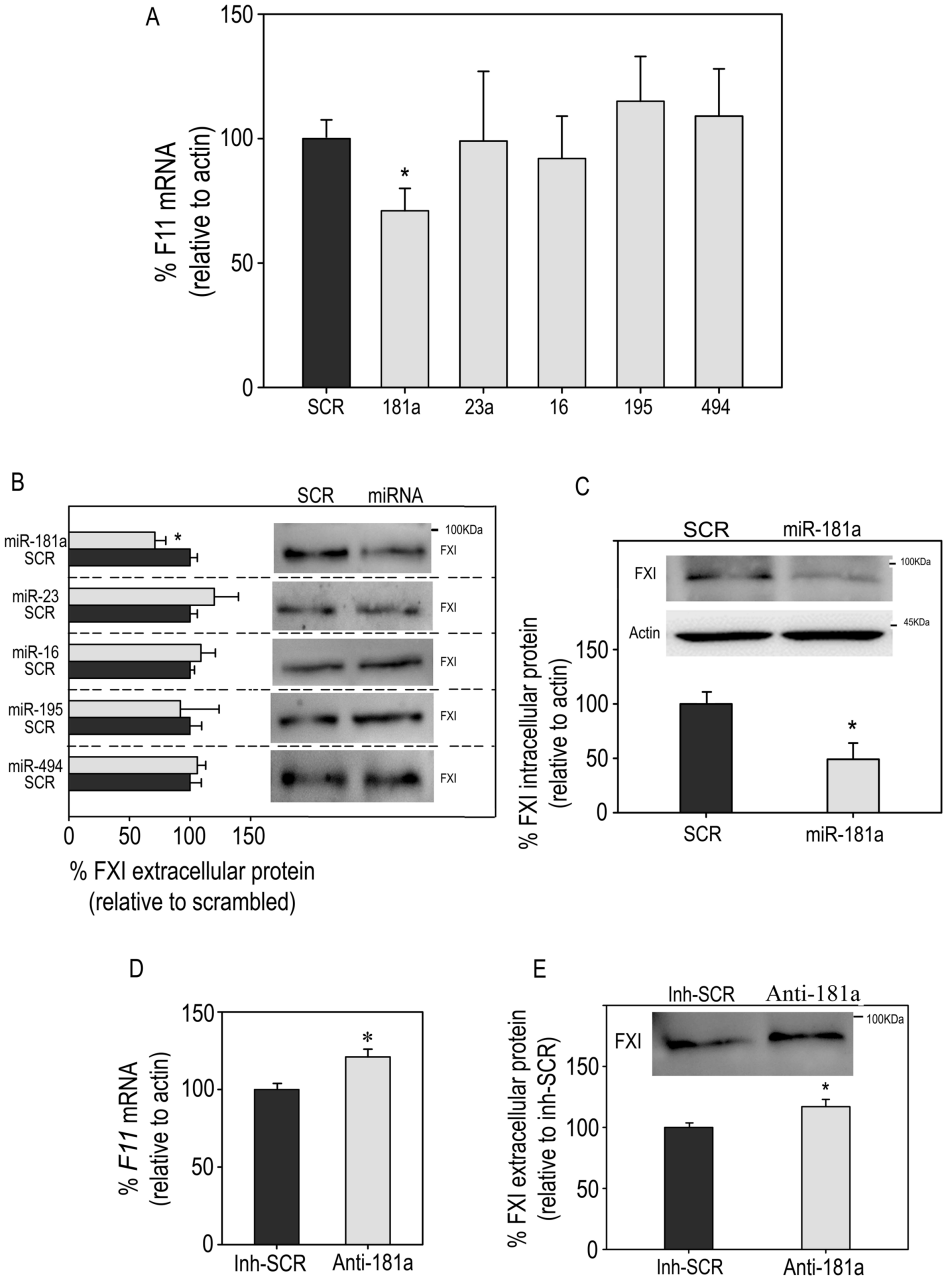
Effect of miRNAs on FXI expression. HepG2 cells were transfected with 100 nM mimic precursors miR-181a-5p (181a), miR-23a-3p (23a), miR-16-5p (16), miR-195-5p (195), miR-494 (494) or SCR. Protein lysate, total RNA and extracellular media were obtained after 48 h incubation and analyzed. (A) qRT-PCR analysis of *F11* mRNA expression. (B) Densitometric analysis of FXI extracellular protein expression with a representative Western blot. (C) Densitometric analysis of FXI intracellular protein expression with a representative Western blot in cells transfected with SCR and 181a. (D) qRT-PCR analysis of *F11* mRNA expression in PLC/PRF/5 cells transfected with 100 nM miR-181a-5p inhibitor or SCR inhibitor. (E) Densitometric analysis of FXI extracellular protein expression with a representative Western blot in PLC/PRF/5 cells transfected with miR-181a-5p inhibitor (anti-181a) or SCR inhibitor (Inh-SCR). Results are represented as mean ± SD of three replicates from three independent experiments. The normalized data were expressed as changes relative to the data of the cells transfected with SCR mimic or SCR inhibitor and set as 100%. *P<0.05. Student's t-test was calculated in mimic precursors *vs*. SCR.

To test which miRNAs may inhibit FXI expression, we employed HepG2 cells, expressing lower levels of these miRNAs than the liver (Figure S1A). Transfection of HepG2 cells with the different miRNA mimics showed that only miR-181a-5p mimic provoked a significant reduction of endogenous *F11* mRNA levels of almost 30% (100% *vs*. 71±9%; p = 0.03; N = 3) compared with non-specific scrambled negative control (SCR) transfection (Figure 2A). No inhibition was found when transfecting HepG2 with the other selected miRNAs or with miR-494 (Figure 2A). In fact, miR-181a-5p caused a significant decrease (∼30%) in the levels of extracellular FXI (100% *vs*. 71±7%; p = 0.04; N = 3) (Figure 2B).

In addition, when the intracellular levels of FXI in cells transfected with miR-181a-5p were evaluated an almost 50% decrease was observed compared with cells transfected with SCR (100% *vs.* 53±16%; p = 0.02; N = 3) (Figure 2C).

Next, we tested the effect of a miR-181a-5p inhibitor in another hepatic cell line where miR-181a-5p expression levels were similar to those reported in the liver (Figure S1B), PLC/PRF/5 hepatic cell line. Our results indicated that inhibition of miR-181a-5p increased both the levels of *F11* mRNA (100% *vs.* 121±3%; p = 0.006; N = 3) (Figure 2D) and of extracellular FXI (100% *vs.* 116±6%; p = 0.038; N = 3) (Figure 2E) further supporting a physiological regulation of FXI by miR-181a-5p.

**Figure 3:**
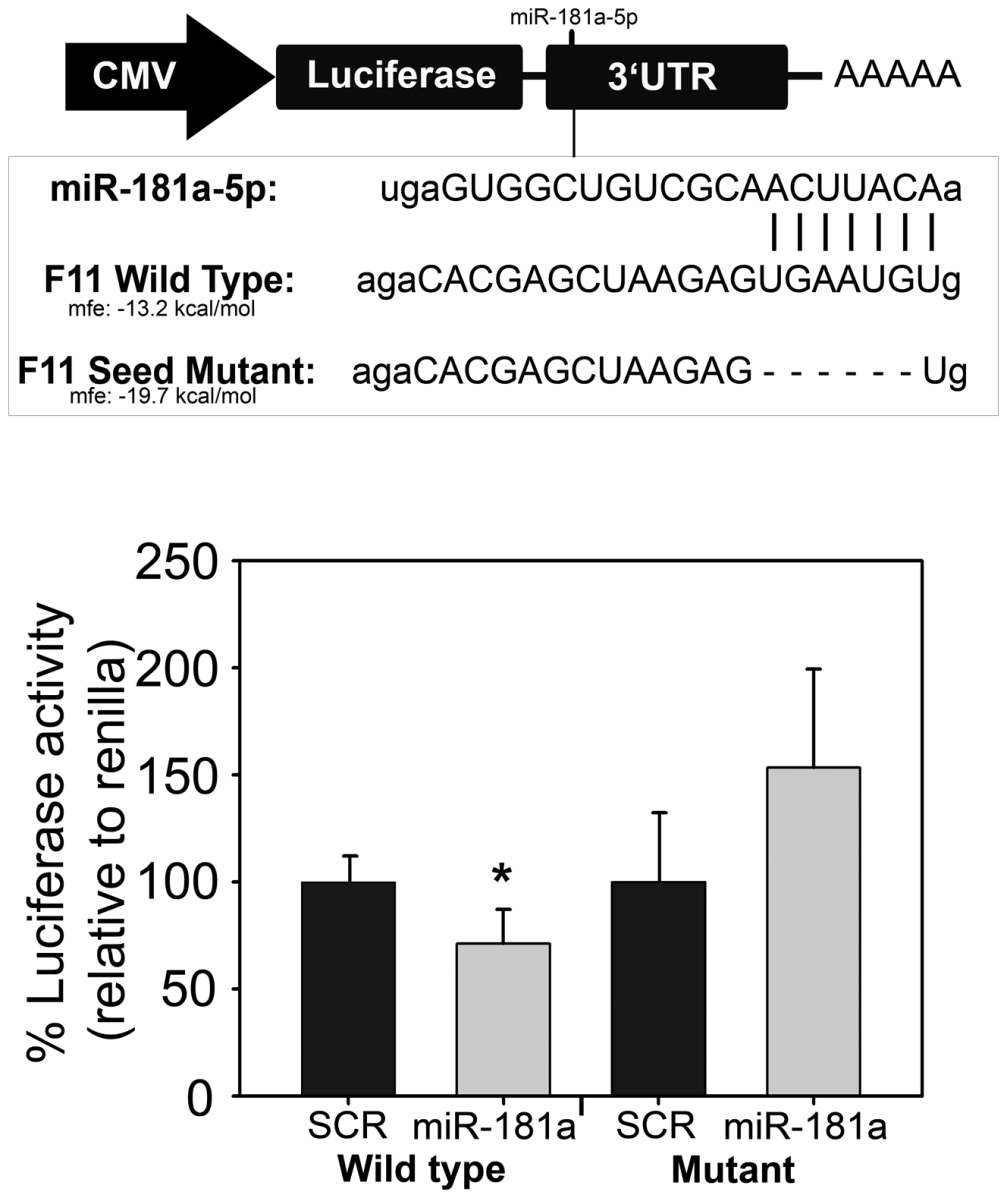
Luciferase reporter assays. Schematic diagram of the luciferase reporter plasmids including *F11* WT 3′UTR or *F11* mutant 3′UTR in which the seven nucleotides forming the seed region of miR-181a-5p were deleted. HCT-DK cells, that do not express miR-181a-5p nor other Dicer-dependent miRNAs that may interfere in miRNA overexpression experiments, were transfected with either *F11* WT 3′UTR or *F11* mutant 3′UTR along with 100 nM miR-181a-5p precursor. A SCR precursor was used as control. Luciferase activities were normalized to renilla activities. Results are represented as mean ± SD of three replicates from three independent experiments. The normalized data were expressed as changes relative to the data of the cells transfected with SCR mimic and set as 100%. *P<0.05. Student's t-test was calculated in mimic precursors vs. SCR.

Whether the lower levels of *F11* mRNA observed in transfected HepG2 were due to an indirect effect of miR-181a-5p or to mRNA decay was further investigated. Co-transfection of HCT-DK cells with a luciferase reporter vector containing the 3′UTR of *F11* and miR-181a-5p showed a significant decrease of ∼30% of the luciferase activity in comparison with SCR (100±17% *vs*. 71±27%; p = 0.04; N = 3). This inhibition was not observed when using a mutated vector which lacked the binding site of miR-181a-5p (Figure 3).

### *F11* mRNA and FXI levels were inversely correlated with miR-181a-5p in human livers

**Figure 4:**
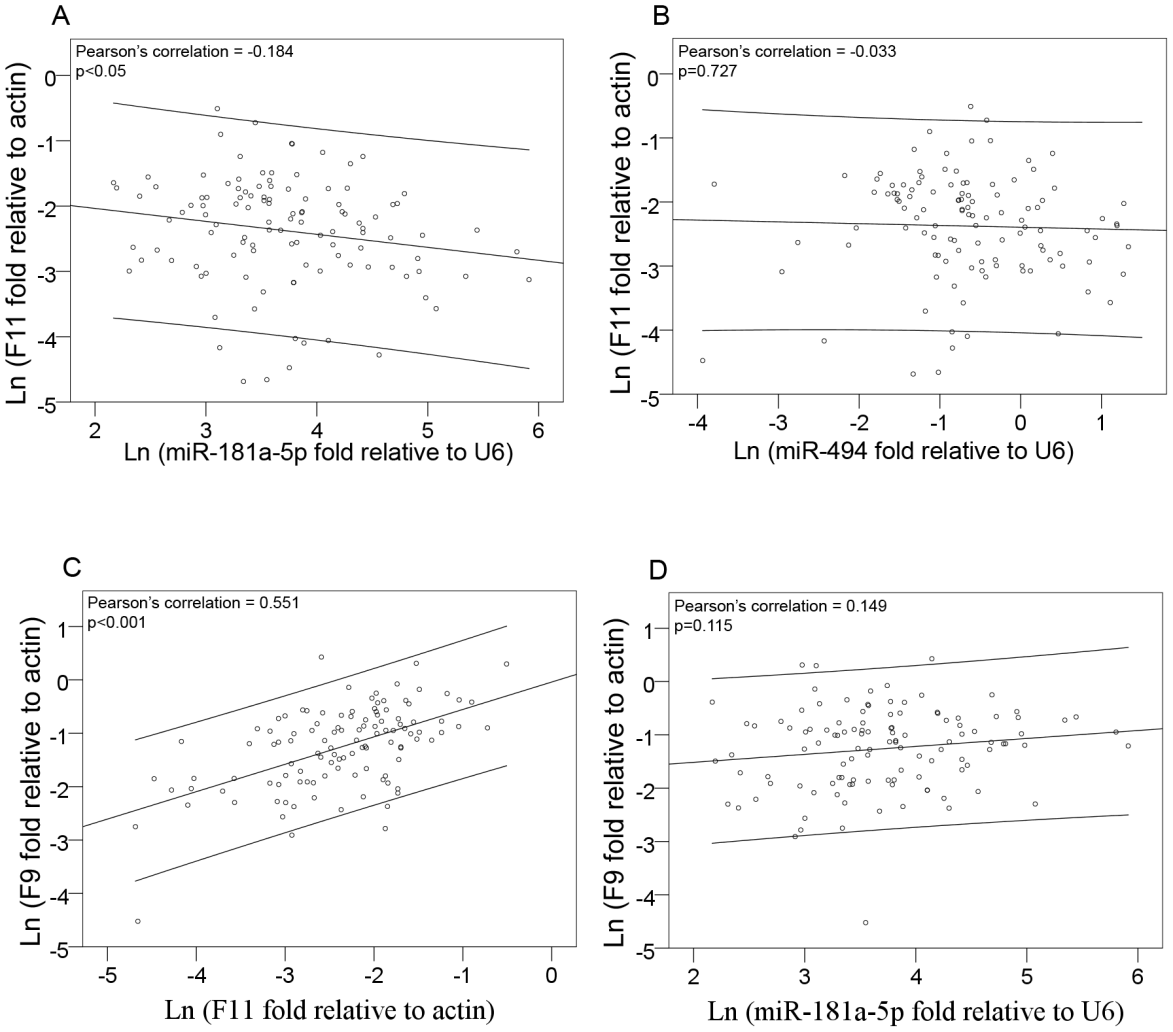
*Ex vivo* expression of *F11* mRNA and mature miRNAs. Linear regression analysis between endogenous mature miRNAs (miR-181a-5p and miR-494) levels and *F11* mRNA (A & B respectively). (C) Linear regression between *F11* and *F9* mRNAs and (D) between miR-181a-5p and *F9* mRNA. qRT-PCR were performed in total RNA purified from healthy livers (n = 114). Statistical significance was taken as p<0.05. The results are presented as Ln fold change with respect to the normalization standard.

In order to establish the potential physiological significance of the above results, we measured *F11* mRNA and miR-181a-5p levels in samples from a cohort of healthy livers that had been used for liver transplant. Using a linear regression model, *F11* mRNA levels were found to be inversely and significantly related to miR-181a-5p levels (r = −0.184; p<0.05) (Figure 4A). To confirm the specificity of this result, we compared levels of *F11* mRNA with those of miR-494 (that showed no *in silico* or *in vitro* effect on FXI expression) and we found no association between these two molecules (r = −0.033; p = 0.727) (Figure 4B).

Previous studies have shown that FXI plasma antigenic levels are correlated with those of FIX [2]. In this study, we observed a strong correlation between *F9* and *F11* mRNA levels (Figure 4C), which supported the described correlation in plasma. Aiming to further confirm the specificity of *F11* mRNA: miR-181a-5p interaction, we investigated a potential correlation between miR-181a-5p and *F9* mRNA levels. As expected, our results demonstrated that miR-181a-5p had no influence on *F9* mRNA levels in healthy livers (p = 0.115) (Figure 4D).

**Figure 5:**
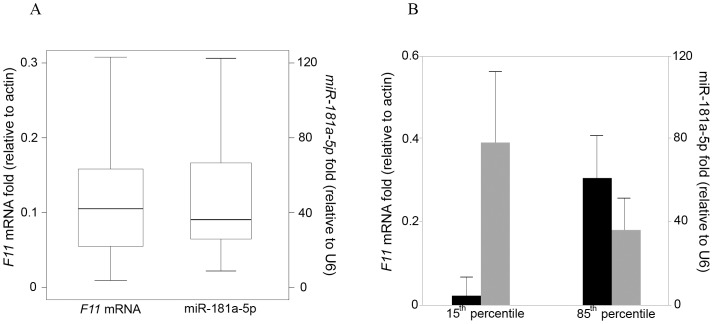
Expression range of miR-181a-5p and *F11* mRNA in healthy livers. (A) Box plot of *F11* mRNA and miR-181a-5p levels in livers. The upper and lower bars are the 90^th^ and 10^th^ percentiles, respectively. (B) Levels of miR-181a-5p (black) in livers corresponding to 15^th^ and 85^th^ percentiles of *F11* mRNA expression (grey), mean ± SD.

Next, we investigated the dynamic range of expression of *F11* mRNA in hepatocytes, finding that it was larger than that described for plasma levels of FXI [26]. More specifically, there was a three-fold difference in *F11* mRNA values between the 75^th^ and 25^th^ percentiles (Figure 5A). Interestingly, we also observed a wide range of expression of miR-181a-5p among individuals. In fact, miR-181a-5p expression in liver samples with *F11* mRNA levels below 15^th^ percentile was two-fold higher than in the samples above the 85^th^ percentile (Figure 5B).

## Discussion

It has been consistently reported that miRNAs may regulate haemostatic proteins such as fibrinogen [22], tissue factor [23], PAI-1[24] or antithrombin [21], while variations in the levels of miRNAs [23] or in the efficacy of the miRNA: mRNA interaction [27] might have an impact on the development of thrombotic diseases. In the present study, we provide new evidence that miRNAs fine-regulates the expression of FXI in human hepatocytes, which in turn opens an alternative regulation pathway that may be exploited for future studies.

The *in silico* results were the starting point in our work. Bearing in mind that approximately 70% of all *in silico* predictions are thought to be false-positive [28], this encouraged us to more accurately establish filters to select the candidate miRNAs to be studied. Our filters led to the selection of 4 candidate miRNAs with potential to bind to *F11* mRNA and, even then, we observed 75% false positives. The effective prediction of miRNA: mRNA pairs in animal systems is still a challenge because of the complexity of this interaction [29]. Future studies to unravel these issues will probably help identify a more selective *in silico* search method for additional miRNAs which target FXI and other haemostatic factors.

In a second step consisting in performing an *in vitro* validation, only miR-181a-5p was potentially a direct regulator of *F11* expression, which enabled it to induce a significant decrease in both extra- and intracellular FXI and *F11* mRNA levels. As expected, and in accord with other studies describing that the effect of a given miRNA on its target is generally modest [19], [20], [28] and that it is the total of various miRNA interactions that would determine the effect as a whole, we found that the effect of miR-181a-5p on FXI expression was mild. Future studies will determinate additional miRNAs that may act in conjunction with miR-181a-5p to regulate FXI expression. In this sense, we found that a neutral miRNA (miR-494) neither interacted nor correlated with *F11* mRNA (Figure 4B). Moreover, the lack of correlation found between miR-181a-5p and *F9* mRNA further supported our hypothesis that miR-181a-5p has a specific effect on FXI expression.

Next, we investigated the physiological relevance of FXI regulation by miR-181a-5p. Given our inability to test this hypothesis *in vivo*, we performed *ex vivo* analysis in livers from healthy donors that had been used for transplant. Our data showed that levels of miR-181a-5p and *F11* mRNA were correlated in human livers (Pearson's coefficient  = −0.184, p<0.05). In fact, the *in vitro* study demonstrated that the decrease of both target *F11* mRNA and protein levels was proportional and therefore we speculate that the *ex vivo* correlation may be extrapolated. Together with the fact that protein levels are determined by additional tightly modulating processes including protein degradation rates, the final effect of a miRNA on an mRNA is very difficult to predict [30]. In this sense, we found a three-fold difference in *F11* mRNA values between the 75^th^ and 25^th^ percentiles in healthy livers, whereas only a 1.3-fold difference in plasma between the same percentiles has previously been found [26]. Therefore, translational, post-translational, and degradation processes, together with the regulation of the secretory pathway (involving both miRNAs and target genes), may act to adjust the final amount of FXI in plasma.

Many observations of miRNA-mediated regulation in mammalian cells considered them as fine-tuners of gene expression [31]. Indeed, miRNA expression can be regulated by the same genetic alterations that modulate protein coding genes [32] as well as by environmental factors [6]. Specifically, miR-181a-5p has been shown to be regulated by dopamine [33] and TGF-β [34]. Our data showed a surprising heterogeneity of miR181a-5p expression in human liver samples (Figure 5A). We observed a 2.5-fold difference in miRNA values between the 75^th^ and 25^th^ percentiles, which reached more than 12-fold when the 90^th^ and 10^th^ percentiles were compared. Interestingly, recently Mendell and Olson suggested that miRNA deficiency or overexpression may have an important impact under pathophysiological stress conditions [35] while, in physiological conditions, miRNAs may only play a modest role in regulating their target.

Overall, our *in vitro* and *ex vivo* results establish miRNAs as new modulators of FXI, opening up new prospects for the regulation of FXI by miRNAs that deserves further attention and confirmation. However, our *in vitro* experimental conditions did not allow us to test the effect of miR-181a-5p on functional activity of FXI. Additional studies are necessary to fully understand this new FXI regulation and to test the possibility that it is regulated by other miRNAs in an indirect way. It would also be useful to further investigate the association of miR-181a-5p expression with the development of thromboembolic disease. In this sense, FXI is seen as a potential therapeutic target since its inhibition prevents thrombosis without bleeding episodes [10]. Indeed, the use of several miRNA-based therapies in the liver is under investigation. The most advanced is the use of anti-miR-122 in the treatment of hepatitis C [36]. On the other hand, it has already been shown that antisense oligonucleotides inhibit factor XI expression *in vivo* [37], and a clinical phase 2 trial is currently ongoing (http://www.isispharm.com/Pipeline/Therapeutic-Areas/Cardiovascular.htm#ISIS-FXIRx) using a drug antisense in patients with total knee arthroplasty. Therefore, the characterization of miR-181a-5p that regulates FXI expression may be seen as an opportunity to start envisaging the potential use of miRNA precursors as an anti-thrombotic drug.

## Materials and Methods

### Cell line and tissue samples

HepG2 (American Type Culture Collection, Manassas, VA), PLC/PRF/5 (kind gift of Fernando Corrales, CIMA, Pamplona, Spain. Original commercial source: American Type Culture Collection, Manassas, VA), and human colon cancer cell line deficient for Dicer [38], [39] (HCT-DK) were conventionally cultured. Briefly, HepG2 and PLC/PRF/5 were cultured in DMEM (Life Technologies, Madrid, Spain) and HCT-DK in McCoy's 5A (Sigma-Aldrich, Madrid, Spain). All media were supplemented with 0.1mM non-essential amino acids, 2 mM Glutamax I, and with 10% fetal calf serum (Life Technologies, Madrid, Spain). Cells were grown at 37°C under 5% CO_2_. Liver samples from Caucasian donors (n = 114) were kindly provided by the Biobanc CIBERehd [40] (La Fe Hospital, Valencia, Spain) (n = 19) and by St. Jude Children's Research Hospital Liver Resource (Liver Tissue Procurement and Distribution System (NIH Contract #N01-DK-9-2310) and the Cooperative Human Tissue Network) [41] (n = 95) (Table S2). All donors gave a written informed consent that was recorded following the procedures of each Biobanc. Human liver studies were further approved by Local Ethics Committee from Hospital Universitario Morales Meseguer in Murcia (#ESTU-19/12).

### MiRNA array and *in silico* identification of miRNA binding sites in *F11* mRNA

To identify miRNAs expressed in healthy liver, we performed expression arrays including 1,898 human mature miRNAs (LCSciences, Houston, TX; Sanger mirBase Release 18.0 and 19.0) using total RNA extracted from 4 healthy human liver samples (Table S1). Raw miRNA microarray data are available in public archives under corresponding author name (GSE61219). For the determination of mature hepatic miRNAs potentially regulating human *F11* 3′UTR, we used four miRNA prediction algorithms.

### Cell transfection

To validate *in silico* experiments, we performed transfection assays in cell lines mentioned above. Briefly, cells were seeded twenty four hours before transfection in complete medium without antibiotics and transfected with 100 nM of chemically modified double-stranded RNAs that mimic endogenous miRNAs (mimic), 100 nM miRNA inhibitors (miRCURY LNA microRNA Inhibitor from Exiqon, Vedbaek, Denmark) or 100 nM SCR from Life Technologies (Madrid, Spain) as previously described [40]. Transfection efficiency was >90% (Figure S2). After 48 h, supernatants and cells were collected for subsequent mRNA and protein analyses.

### MiRNA and mRNA expression levels

Total RNA was isolated from both fresh livers and transfected cells using Trizol Reagent (Life Technologies, Madrid, Spain). RNA (400 ng) and SuperScript III First-Strand Synthesis System (Life Technologies, Madrid, Spain) were used for reverse transcription (RT) reactions. *F11* and *ACTB* (as endogenous reference control) genes expression were quantified by qRT-PCR (Hs01030011_m1 and Hs99999903_m1, respectively, from Life Technologies, Madrid, Spain).

Commercial assays for miR-181a-5p, miR-23a-3p, miR-16-5p, miR-195-5p, miR-494, and U6 snRNA (endogenous reference control) (Life Technologies, Madrid, Spain) were used to quantify expression levels of miRNAs in human cell lines and/or hepatocytes.

### Western blot

Proteins from the lysate of transfected HepG2 cells (60 µg) or liver (50 µg) were blotted and immunostained with anti-human FXI polyclonal antibody (Enzyme Research Laboratories, Swansea, UK) and anti-human β-actin monoclonal antibody (Sigma-Aldrich, Madrid, Spain). Additionally, we collected and lyophilized 500 µL supernatants from HepG2 and PLC/PRF/5 using CentriVap Concentrator (Labconco, Kansas, MO) and 24 µL were blotted and immunostained with anti-human FXI polyclonal antibody. FXI and β-actin were immunodetected with the appropriate secondary antibody labeled with peroxidase (GE Healthcare, Barcelona, Spain). Detection was performed using ECL Prime Western Blotting Detection Kit (GE Healthcare, Barcelona, Spain) and ImageQuant LAS 4000 Imager. Densitometric analysis was performed with ImageJ software (http://rsb.info.nih.gov/ij/). Data were expressed as changes relative to the values of the cells transfected with SCR, taken as 100%.

### Luciferase reporter assay

In order to test if the regulation of F11 by miRNAs is done through a direct interaction between both molecules, we performed luciferase reporter assays, as described below.

#### Plasmid construction

PCR product (1,060 bp) containing the F11 3′UTR from human genomic DNA (NM_000128), obtained using primers *F11*-3′UTR_F and *F11*-3′UTR_R, was cloned into the pCR 2.1 vector (Life Technologies, Madrid, Spain) (Table S3). Positive clones were digested with SpeI and MluI (New England Biolabs, Ipswich, MA) and the insert was subcloned into luciferase reporter plasmid pMIR-REPORT (Life Technologies, Madrid, Spain) previously digested with SpeI and MluI. Insertion of *F11* 3′UTR was checked by sequencing (ABI3130 XL, Life Technologies Corporation, Carlsbad, CA). All sequence analyses and alignments were performed with the SeqmanPro program (Lasergene version 7.1, DNASTAR, Madison, WI).

To generate mutations in the predicted target site for miR-181a-5p, seven nucleotides (TGAATGT) located in the seed sequence were deleted using the QuikChange site-directed mutagenesis kit (Agilent Technologies, Santa Clara, CA). *In silico* prediction, using RNAHybrid, of miR-181a-5p binding to the mutated sequence showed a 33% decrease in the minimum free energy value (not shown) indicating that miR-181a-5p: *F11* mRNA interaction was completely suppressed. Sequencing was performed to check for the deletion of the seed sequence. The primers used (del_181_AS and Del_181_S) are detailed in Table S3.

#### Luciferase vector transfection

HCT-DK cells, that do not express miR-181a-5p, were seeded at a density of 80,000 cells/well in 24-well plates with complete McCoy's 5A supplemented with 10% fetal calf serum without antibiotics. The following day, cells were co-transfected with miR-181a-5p (both pMIR-REPORT plasmids -1000 ng/well- wild type or mutated for the miR-181a-5p seed site) or SCR precursor, and 100 ng/well of renilla luciferase control plasmid (pRL-TK; Promega, Madison, WI) using Lipofectamine LTX (Life Technologies,Madrid, Spain) according to the manufacturer's instructions. Luciferase assays were performed as previously described [40].The enzymatic activities of renilla and firefly luciferases were quantified in a Synergy 2 luminometer (Biotek, Winooski, VT). Each combination of pMIR-REPORT (wild-type and mutated 3′UTR) and pRL-TK was tested in triplicate in five independent experiments. Firefly luciferase activity was normalized to renilla luciferase activity for each transfected well. The normalized data were expressed as changes relative to the data of the cells transfected with 100 nM miR-181a-5p mimic, SCR was taken as 100%.

### Statistical Analysis

Comparisons between groups were performed by the unpaired t-test. Data are given as mean ± SD. Linear regression tests were performed; β regression coefficient (r) and r^2^ were calculated. Results were considered statistically significant for p<0.05. Analyses were carried out using Statistical Package for Social Science (version 21.0; SPSS, Chicago, IL).

## Supporting Information

Figure S1 **Levels of miRNAs in human liver and cell lines.** Levels of miRNAs were Quantified by qRT-PCR. (A) Levels of miRNAs in HepG2 relative to human liver. (B) Levels of miR181a-5p in HepG2 and PLC/PRF/5 relative to human liver. Results are represented as mean ± SD of three replicates from two independent experiments.
(TIF)

Figure S2 **miRNA inhibitor transfection efficiency.** miRCURY LNA microRNA Inhibitor Negative Control (100 nM) labeled with fluorescein (Exiqon, Vadbaek, Denmark) were transfected into PLC/PRF/5 cells with siPORTTM NeoFXTM (Life TechnologiesTM, Madrid, Spain), following manufacturer's instructions. After 6 hours transfection, cells were harvested and washed with PBS. Flow cytometry was performed using a BD FACSCalibur flow cytometer (BD Biosciences, Madrid, Spain) and samples were run through the flow cytometer until 2,000 events were collected. The mean ± SD of transfection efficiency for three replicates was 92.1%±0.3%. X-axis represents the intensity of fluorescence for FL1 channel in log scale and Y-axis the numbers of cells. We defined transfection efficiency as a percentage of cells positive for FL1 (dotted lines), taken as background signal the non-LNA transfected cells signal (solid lines).
(TIF)

Table S1 **MiRNA expression in human liver.**
(DOCX)

Table S2 **Characteristics of liver donors.**
(DOCX)

Table S3 **Primers used in the study.**
(DOCX)
