# Supplementary figures and images for: Regulation of Coagulation Factor XI Expression by MicroRNAs in the Human Liver
Source: PLoS One. 2014 Nov 7;9(11):e111713. doi: 10.1371/journal.pone.0111713 (PMC4224396; doi:10.1371/journal.pone.0111713)

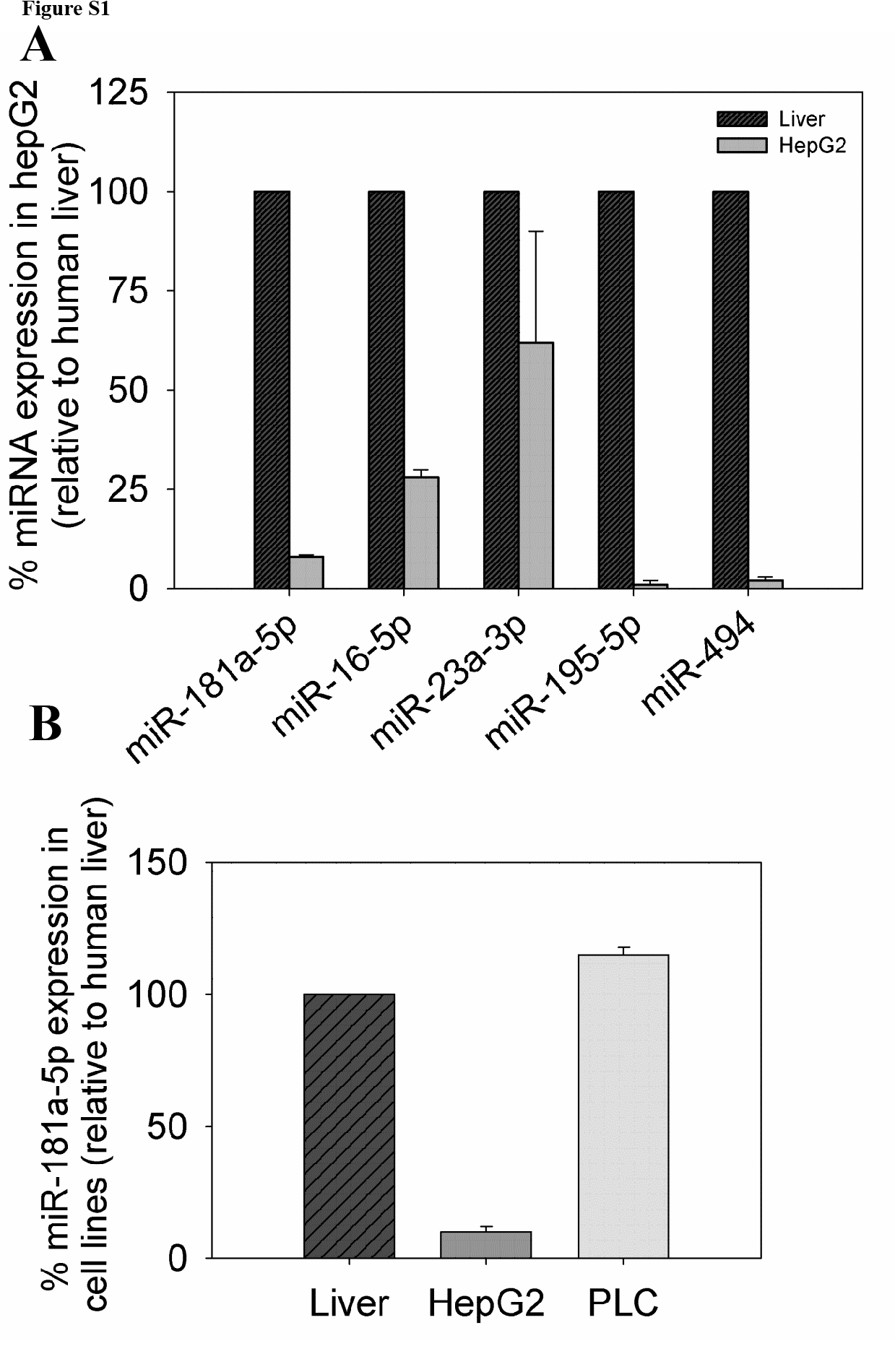

Supplement: Figure S1 — Levels of miRNAs in human liver and cell lines. Levels of miRNAs were Quantified by qRT-PCR. (A) Levels of miRNAs in HepG2 relative to human liver. (B) Levels of miR181a-5p in HepG2 and PLC/PRF/5 relative to human liver. Results are represented as mean ± SD of three replicates from two independent experiments. (TIF) [file pone.0111713.s001.tif]

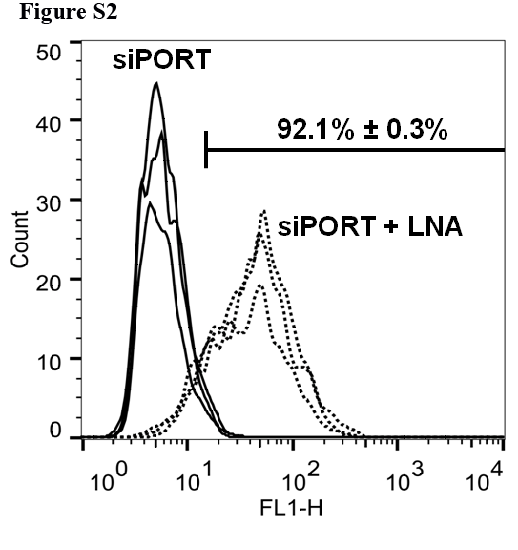

Supplement: Figure S2 — miRNA inhibitor transfection efficiency. miRCURY LNA microRNA Inhibitor Negative Control (100 nM) labeled with fluorescein (Exiqon, Vadbaek, Denmark) were transfected into PLC/PRF/5 cells with siPORTTM NeoFXTM (Life TechnologiesTM, Madrid, Spain), following manufacturer's instructions. After 6 hours transfection, cells were harvested and washed with PBS. Flow cytometry was performed using a BD FACSCalibur flow cytometer (BD Biosciences, Madrid, Spain) and samples were run through the flow cytometer until 2,000 events were collected. The mean ± SD of transfection efficiency for three replicates was 92.1%±0.3%. X-axis represents the intensity of fluorescence for FL1 channel in log scale and Y-axis the numbers of cells. We defined transfection efficiency as a percentage of cells positive for FL1 (dotted lines), taken as background signal the non-LNA transfected cells signal (solid lines). (TIF) [file pone.0111713.s002.tif]
