# Supplementary material for: Regulation of Coagulation Factor XI Expression by MicroRNAs in the Human Liver
Source: PLoS One. 2014 Nov 7;9(11):e111713. doi: 10.1371/journal.pone.0111713 (PMC4224396; doi:10.1371/journal.pone.0111713)
Supplement: Table S1 — MiRNA expression in human liver. (DOCX) [file pone.0111713.s003.docx]

**Table S1: MiRNA expression in human liver.** MiRNA arrays (n=4) using Sanger miRBase Release 18.0 and 19.0 revealed a significant expression of 219 miRNAs (significant signal intensities considered with p-values < 0.05). The sample signal median from the three independent arrays for each miRNA is shown.

| **Index** | **Human miRNA** | | **Sample Signal**  **(arbitrary units)** | **Index** | | **Human miRNA** | **Sample Signal**  **(arbitrary units)** | **Index** | **Human miRNA** | **Sample Signal**  **(arbitrary units)** |
| --- | --- | --- | --- | --- | --- | --- | --- | --- | --- | --- |
| 1 | *miR-122-5p* | *28,636* | | 74 | *miR-4530* | | *1,001* | 147 | miR-374b-5p | 227 |
| 2 | *miR-26a-5p* | *14,457* | | 75 | *miR-3607-5p* | | *976* | 148 | miR-99b-5p | 226 |
| 3 | *let-7f-5p* | *10,867* | | 76 | *miR-22-5p* | | *966* | 149 | miR-378f | 226 |
| 4 | *let-7a-5p* | *10,749* | | 77 | *miR-1973* | | *962* | 150 | miR-378c | 217 |
| 5 | *miR-30b-5p* | *9,297* | | 78 | *miR-4281* | | *933* | 151 | miR-4532 | 215 |
| 6 | *let-7d-5p* | *8,559* | | 79 | *miR-378e* | | *907* | 152 | miR-193a-3p | 215 |
| 7 | *miR-23b-3p* | *8,276* | | 80 | *miR-320c* | | *903* | 153 | miR-3591-3p | 210 |
| 8 | *miR-4443* | *8,147* | | 81 | *miR-4324* | | *891* | 154 | miR-1275 | 208 |
| 9 | *miR-3960* | *7,402* | | 82 | *miR-19b-3p* | | *888* | 155 | miR-130b-3p | 207 |
| 10 | *miR-192-5p* | *7,183* | | 83 | *miR-5001-5p* | | *873* | 156 | miR-5100 | 202 |
| 11 | *miR-3665* | *6,889* | | 84 | *miR-4472* | | *813* | 157 | miR-4284 | 201 |
| 12 | *miR-1273g-3p* | *6,692* | | 85 | *miR-378d* | | *739* | 158 | miR-128 | 193 |
| 13 | *miR-214-3p* | *6,628* | | 86 | *miR-25-3p* | | *729* | 159 | miR-30b-3p | 191 |
| 14 | *miR-30c-5p* | *6,448* | | 87 | *miR-27a-3p* | | *718* | 160 | miR-151a-3p | 190 |
| 15 | *let-7c* | *6,292* | | 88 | *miR-466* | | *712* | 161 | miR-193a-5p | 185 |
| 16 | ***miR-23a-3p*** | ***6,052*** | | 89 | *miR-320d* | | *669* | 162 | miR-3609 | 178 |
| 17 | *miR-26b-5p* | *5,925* | | 90 | *miR-320e* | | *664* | 163 | miR-30c-1-3p | 175 |
| 18 | *miR-194-5p* | *5,007* | | 91 | *miR-5096* | | *664* | 164 | miR-331-3p | 173 |
| 19 | *let-7g-5p* | *4,837* | | 92 | *miR-191-5p* | | *651* | 165 | miR-4485 | 172 |
| 20 | *miR-4787-5p* | *4,637* | | 93 | *miR-17-5p* | | *643* | 166 | miR-4286 | 172 |
| 21 | *miR-451a* | *3,993* | | 94 | *miR-139-5p* | | *619* | 167 | miR-4534 | 162 |
| 22 | *miR-125b-5p* | *3,952* | | 95 | *miR-1246* | | *617* | 168 | miR-4778-5p | 158 |
| 23 | *let-7b-5p* | *3,866* | | 96 | *miR-4508* | | *610* | 169 | miR-101-3p | 155 |
| 24 | *miR-148a-3p* | *3,747* | | 97 | *miR-4734* | | *608* | 170 | miR-340-5p | 151 |
| 25 | *miR-1260b* | *3,548* | | 98 | *miR-3178* | | *605* | 171 | miR-3195 | 151 |
| 26 | *miR-145-5p* | *3,522* | | 99 | *miR-320b* | | *602* | 172 | miR-4505 | 150 |
| 27 | ***miR-16-5p*** | ***3,513*** | | 100 | *miR-4459* | | *595* | 173 | miR-142-5p | 145 |
| 28 | *miR-24-3p* | *3,487* | | 101 | *miR-29c-3p* | | *593* | 174 | miR-375 | 139 |
| 29 | *miR-29a-3p* | *3,419* | | 102 | *miR-4466* | | *580* | 175 | miR-3607-3p | 138 |
| 30 | *miR-146a-5p* | *3,212* | | 103 | *miR-152* | | *579* | 176 | miR-29c-5p | 127 |
| 31 | *miR-92a-3p* | *3,147* | | 104 | *miR-106a-5p* | | *544* | 177 | miR-197-3p | 120 |
| 32 | ***miR-195-5p*** | ***3,046*** | | 105 | *miR-130a-3p* | | *542* | 178 | miR-4484 | 120 |
| 33 | *miR-1915-3p* | *2,943* | | 106 | *miR-378g* | | *536* | 179 | miR-1268b | 115 |
| 34 | *miR-221-3p* | *2,885* | | 107 | miR-223-3p | | 499 | 180 | miR-193b-3p | 112 |
| 35 | *miR-146b-5p* | *2,845* | | 108 | miR-22-3p | | 487 | 181 | miR-3676-5p | 104 |
| 36 | *miR-30a-5p* | *2,845* | | 109 | miR-10a-5p | | 486 | 182 | miR-181b-5p | 102 |
| 37 | *miR-638* | *2,830* | | 110 | miR-20a-5p | | 483 | 183 | miR-192-3p | 99 |
| 38 | *miR-99a-5p* | *2,598* | | 111 | miR-376c | | 479 | 184 | miR-200a-3p | 93 |
| 39 | *miR-4516* | *2,580* | | 112 | miR-148b-3p | | 478 | 185 | miR-497-5p | 90 |
| 40 | *miR-103a-3p* | *2,514* | | 113 | miR-361-5p | | 467 | 186 | miR-454-3p | 80 |
| 41 | *miR-215* | *2,418* | | 114 | miR-126-3p | | 466 | 187 | miR-484 | 80 |
| 42 | *miR-122-3p* | *2,357* | | 115 | miR-29b-3p | | 458 | 188 | miR-127-3p | 79 |
| 43 | *miR-199a-3p* | *2,320* | | 116 | miR-4454 | | 456 | 189 | miR-200b-3p | 78 |
| 44 | *miR-30d-5p* | *2,221* | | 117 | miR-425-5p | | 445 | 190 | miR-345-5p | 76 |
| 45 | *miR-107* | *2,193* | | 118 | miR-423-5p | | 438 | 191 | miR-377-3p | 72 |
| 46 | *miR-30e-5p* | *2,148* | | 119 | miR-335-5p | | 422 | 192 | miR-1307-3p | 71 |
| 47 | *miR-143-3p* | *2,130* | | 120 | miR-186-5p | | 400 | 193 | miR-532-5p | 71 |
| 48 | *let-7i-5p* | *2,062* | | 121 | miR-155-5p | | 399 | 194 | miR-4492 | 69 |
| 49 | *miR-27b-3p* | *1,769* | | 122 | miR-98 | | 377 | 195 | miR-885-3p | 69 |
| 50 | *miR-100-5p* | *1,736* | | 123 | miR-3940-5p | | 369 | 196 | miR-148a-5p | 67 |
| 51 | *miR-125a-5p* | *1,643* | | 124 | miR-4463 | | 366 | 197 | miR-487b | 67 |
| 52 | *miR-574-3p* | *1,556* | | 125 | miR-644b-3p | | 357 | 198 | miR-1469 | 66 |
| 53 | *miR-3656* | *1,555* | | 126 | miR-4298 | | 349 | 199 | miR-378a-5p | 64 |
| 54 | *miR-151b* | *1,547* | | 127 | miR-4447 | | 342 | 200 | miR-4521 | 64 |
| 55 | *miR-151a-5p* | *1,527* | | 128 | miR-2861 | | 338 | 201 | miR-362-5p | 59 |
| 56 | *miR-494* | *1,395* | | 129 | miR-483-5p | | 326 | 202 | miR-324-5p | 57 |
| 57 | *miR-455-3p* | *1,382* | | 130 | miR-34a-5p | | 315 | 203 | miR-7-1-3p | 55 |
| 58 | *miR-23c* | *1,379* | | 131 | miR-378i | | 307 | 204 | miR-203 | 53 |
| 59 | *miR-150-5p* | *1,308* | | 132 | miR-15a-5p | | 294 | 205 | miR-145-3p | 53 |
| 60 | *let-7e-5p* | *1,264* | | 133 | miR-4739 | | 291 | 206 | miR-361-3p | 49 |
| 61 | *miR-92b-3p* | *1,249* | | 134 | miR-149-3p | | 278 | 207 | miR-500a-3p | 46 |
| 62 | ***miR-181a-5p*** | ***1,233*** | | 135 | miR-424-5p | | 278 | 208 | miR-1182 | 46 |
| 63 | *miR-3196* | *1,222* | | 136 | miR-4707-5p | | 253 | 209 | miR-20b-5p | 37 |
| 64 | *miR-378a-3p* | *1,204* | | 137 | miR-222-3p | | 251 | 210 | miR-425-3p | 33 |
| 65 | *miR-885-5p* | *1,203* | | 138 | miR-28-5p | | 249 | 211 | miR-210 | 32 |
| 66 | *miR-15b-5p* | *1,147* | | 139 | miR-574-5p | | 249 | 212 | miR-455-5p | 32 |
| 67 | *miR-4497* | *1,144* | | 140 | miR-505-3p | | 246 | 213 | miR-660-5p | 29 |
| 68 | *miR-140-3p* | *1,135* | | 141 | miR-3141 | | 245 | 214 | miR-17-3p | 21 |
| 69 | *miR-4488* | *1,098* | | 142 | miR-185-5p | | 239 | 215 | miR-500b | 21 |
| 70 | *miR-342-3p* | *1,083* | | 143 | miR-93-5p | | 237 | 216 | miR-362-3p | 18 |
| 71 | *miR-320a* | *1,078* | | 144 | miR-762 | | 235 | 217 | miR-18a-5p | 18 |
| 72 | *miR-199a-5p* | *1,073* | | 145 | miR-28-3p | | 231 | 218 | miR-363-3p | 15 |
| 73 | *miR-21-5p* | *1,065* | | 146 | miR-106b-5p | | 228 | 219 | miR-18b-5p | 10 |

In bold are shown the selected miRNAs with an expression > 500 arbitrary units. In italics are shown the miRNAs with an expression > 500 arbitrary units.
