# Supplementary material for: Regulation of Coagulation Factor XI Expression by MicroRNAs in the Human Liver
Source: PLoS One. 2014 Nov 7;9(11):e111713. doi: 10.1371/journal.pone.0111713 (PMC4224396; doi:10.1371/journal.pone.0111713)
Supplement: Table S2 — Characteristics of liver donors. (DOCX) [file pone.0111713.s004.docx]

**Table S2. Characteristics of liver donors**

| Characteristics | Liver donors (n=114) |
| --- | --- |
| Age (years)  Mean  Median  Range | 51.0  53.0  2-87 |
| Gender (%M) | 55 |
