# Supplementary material for: Regulation of Coagulation Factor XI Expression by MicroRNAs in the Human Liver
Source: PLoS One. 2014 Nov 7;9(11):e111713. doi: 10.1371/journal.pone.0111713 (PMC4224396; doi:10.1371/journal.pone.0111713)
Supplement: Table S3 — Primers used in the study. (DOCX) [file pone.0111713.s005.docx]

**Table S3. Primers used in the study**

| Oligonucleotide Name | Sequence (5’-3’) |
| --- | --- |
| F11-3’UTR_R | ACGCGTCACTTGATGAATTGTATAGTTG |
| F11-3’UTR_F | GGATTCTGGAGAAAACTCAAGC |
| Del_181_S | GTAGACACGAGCTAAGAGGAAGATAACAGAATTTC |
| del_181_AS | GAAATTCTGTTATCTTCCTCTTAGCTCGTGTCTAC |
